# Range overlap between the sword-billed hummingbird and its guild of long-flowered species: An approach to the study of a coevolutionary mosaic

**DOI:** 10.1371/journal.pone.0209742

**Published:** 2018-12-26

**Authors:** Florencia Soteras, Marcela Moré, Ana C. Ibañez, María del Rosario Iglesias, Andrea A. Cocucci

**Affiliations:** Laboratorio de Ecología Evolutiva y Biología Floral, IMBIV, CONICET, Universidad Nacional de Córdoba, Casilla de Correo, Córdoba, Argentina; Indian Institute of Science, INDIA

## Abstract

The coevolutionary process among free-living mutualists with extremely long matching traits may favor the formation of mutualistic interaction networks through coevolutionary escalation, complementarity and convergence. These networks may be geographically structured; the links among the species of a local network are shaped by the biotic composition of the community, thus creating selection mosaics at broader geographical scales. Therefore, to fully understand a coevolutionary process, it is crucial to visualize the geographical structure of the interaction network across the landscape. In this study we focused on the poorly known interaction system between *Ensifera ensifera* and its guild of long-flowered plant species. We combined occurrence data and environmental variables to predict *E*. *ensifera* distribution, in addition to range polygons available for plant species in order to evaluate the geographical variation in bill length and plant species richness. A positive relationship between bill length and plant species richness within the *E*. *ensifera* range suggests a geographical structuring of the interaction networks. At mid-latitude locations of *E*. *ensifera* range, where hummingbirds attained the longest bills, richness of long-flowered plant species was higher than at low latitude locations. These locations likely represent coevolutionary vortices where long-lasting reciprocal selection probably drove the evolution of long traits, consequently drawing new plant species into the coevolutionary network. Conversely, areas where the sword-billed hummingbird was absent or had shorter bills probably represent coevolutionary coldspots. Our results provide a first insight into this phenotypically specialized plant-pollinator network across the landscape and show candidate areas to test the predictions of the coevolutionary hypothesis, such as reciprocal selection.

## Introduction

For nearly every pollinator guild, species bearing extremely long reward-collecting appendages have evolved. These include a fly [1,2] and a hawkmoth [3–5] with the longest proboscides, a bee with the longest oil-collecting legs [6,7], a bat with the longest tongue [8], and the sword-billed hummingbird (*Ensifera ensifera* Bois.), which bears by far the longest bill among pollinating birds [9]. The appendages of these animals exhibit complementarity with the long flowers of the plants they pollinate. These pollination partnerships represent some of the most conspicuous examples of coevolution and their study has proven to be revealing from the very beginning of evolutionary thinking. Darwin [10] was inspired by the relationship between the extremely long spurred Malagasy star orchid and a long-tongued hawkmoth (whose existence was then predicted) to hypothesize that the extremely long proboscides and flowers resulted from a coevolutionary escalation. He postulated that the underlying mechanism was reciprocal natural selection driven by fitness advantages of both the moths bearing the longest proboscides and the plants bearing the longest flowers. Reciprocal adaptation of interacting species would thus shape trait matching within extreme size ranges [11]. Only in recent times has the reciprocal phenotypic selection postulated by Darwin’s hypothesis been fully demonstrated in the field for a long-tongued fly and long-flowered lily species [2].

These relationships among free-living mutualists often do not consist of pairwise interactions, but rather of species that locally interact with a guild of several partner species. Consequently, the coevolutionary process among free-living mutualists favors the formation of mutualistic networks through coevolutionary complementarity and convergence [12–14]. Several studies have shown that these networks are geographically structured and that the links among species in a local interaction network are shaped by the biotic composition of the community (e.g., [15,16]). The structure of geographically changing interaction networks has the potential to create multispecific selection mosaics composed of communities where selection is reciprocal (“coevolutionary hotspots”) and others where selection is one-sided or absent (“coevolutionary coldspots”). On the one hand, the selection mosaic is expected to produce plant-pollinator networks exhibiting strong dependence among the interaction partners, coevolution and complementarity between reward-collecting traits and flower lengths. On the other hand, there may be communities in which dependence is weak, reciprocal selection is absent and trait complementarity is low. The latter could occur, for instance, at sites where the range of the plant guild exceeds the pollinator range [17].

To our knowledge, the effect of coevolutionary escalation has been focused on free-living antagonists, rather than on free-living mutualists, as a coevolutionary process concurrent with complementarity and convergence. In antagonistic interactions, coevolutionary escalation is expected to drive away species from interaction networks, promoting a higher ecological specialization and eventually pairwise coevolution [13,18]. Contrarily, mutualisms are expected to promote the incorporation of new species into coevolutionary vortices, creating multispecific coevolutionary networks [13,18]. One-sided selection is likely an important process by which new species are drawn into an existing mutualistic network, but escalated reciprocal selection within the core set of mutualists expectedly drives evolution of long traits. Consequently, we should expect coevolutionary hotspots where multiple plant species jointly drive reciprocal selection on their mutualistic partner.

Studies on plant-hummingbird networks have shown that hummingbirds that best match flower length improve nectar intake and that plants whose flowers that best match hummingbird bill lengths benefit from stronger interaction [19,20]. Hence, reciprocal matching leads to fitness benefits for both plants and hummingbirds.In this study we investigated the phenotypically specialized interaction between *E*. *ensifera* and its guild of plants, whose flowers are exclusively pollinated by the sword-billed hummingbird [9,21,22]. Shared coevolutionary history was suggested by Abrahamczyk et al. [23], who showed that the supersection Tacsonia of long-flowered *Passiflora* species has similar divergence time to that of *E*. *ensifera*. Although specific literature on the interaction ecology of this hummingbird and the guild of plants that it pollinates is surprisingly scarce, the sword-billed hummingbird is known to visit a set of long-flowered species [9,24–26]. Since the bill length of *E*. *ensifera* is known to vary geographically [27], long-flowered species are expected to have exerted varying selection pressures across the geographical distribution range of this pollinator species. Therefore, the study of mutualistic networks from a geographical perspective in this system appears promising to detect potential geographical mosaics of coevolution. We expect that reciprocal selection has occurred or is still occurring simultaneously in multiple species in the areas where a coevolutionary vortex has been established. Areas with coevolutionary vortices should be intermingled with areas where reciprocal selection either is not as strong as to have created vortices or is absent. As a first attempt to evaluate the presence of a mosaic of coevolution, differences in the distribution of interacting species across the landscape can be explored (see [28]).

We hypothesized that reciprocal selection would have occurred or may be occurring at sites where *E*. *ensifera* acquired the longest bills, i.e., where coevolutionary escalation has been strongest or most persistent in time, thus representing possible coevolutionary hotspots. Conversely, since short-billed hummingbirds would not be the most efficient pollinators for the long-flowered guild of plants [19,20], coevolutionary coldspots would be represented by areas where *E*. *ensifera* either is absent or did not acquire long bills. Species distribution models (SDMs) can be used to project species range by combining occurrence data and environmental variables [29]. In this study, we developed SDMs for *E*. *ensifera* and used available range polygons of the guild of long-flowered species, to estimate overlapping ranges. Community studies suggest that very long matching traits are shaped by diffuse coevolution, thus pollinator with long appendages interacts more frequently with long-tubed plants [14]. Therefore, we hypothesized that through a combination of coevolutionary escalation, complementarity, and convergence, plant species would be drawn into mutualistic interaction networks over time, creating coevolutionary vortices. Here, reciprocal selection might explain the existence of extraordinarily long traits within the core members of the community. Although this approach cannot substitute field observations of the geographic variations of the interaction networks, it can be used to pinpoint potential areas where to test the plausibility of the postulated hypotheses.

## Materials and methods

### Study system

The sword-billed hummingbird, *E*. *ensifera*, is distributed in the high Andean forests (between 1,300 and 4,500 m a.s.l.) in Venezuela, Colombia, Ecuador, Peru and Bolivia. It exhibits the longest bill among hummingbirds, ranging between 81 and 120 mm in length (mean bill length of females and males: 103 and 96 mm, respectively), nearly as long as the body [9,21,27].

**Fig 1:**
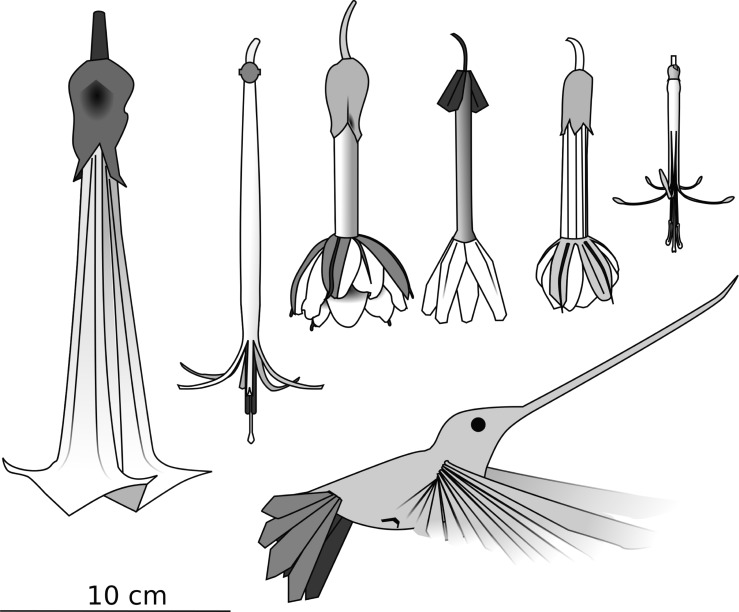
Schematic representation of the guild of plant species pollinated by the sword-billed hummingbird *E*. *ensifera*, from left to right: *Brugmansia sanguinea*, *Aetanthus dichotomus*, *Passiflora mixta*, *P*. *cumbalensis*, *P*. *tripartita* var. *mollisima*,and *Tristerix grandiflorus*.

Flower traits, such as corolla length, have been used to determine the potential set of plant species pollinated by *E*. *ensifera*, since no other hummingbird pollinator in its Neotropical communities would be able to pollinate the deepest flowers (i.e. longer than 80 mm, [19]). We compiled published evidence of *E*. *ensifera* interaction with long-flowered plant species. First, we explored available floras of the mentioned countries searching for flowers longer than 80 mm that had additional bird pollination traits (flower color and time of flowering) to differentiate them from similarly long hawkmoth-pollinated species. The threshold of 80 mm was chosen as a conservative limit of inclusion, since this is the minimum bill length of *E*. *ensifera*. We also searched for long-flowered species among the specimens deposited in different herbaria (Universidad Nacional Mayor de San Marcos, New York Botanical Garden and Museo Botánico de Córdoba). These procedures resulted in a list of 24 species with mean corolla lengths from 95.59 to 146 mm potentially pollinated by the sword-billed hummingbird (Fig 1, Table 1, S1 Fig). Then we searched for Google Scholar articles containing the words “ensifera” and “hummingbird” or “sword-bill*”. We checked that these articles included field observations or anecdotal records of plants being visited by the sword-billed hummingbird (Table 1). Following this procedure, in addition to personal field observations, and study reports of the interaction [22–24,26,30–34], we compiled 24 plant species as the “plant guild” of *E*. *ensifera* (Table 1).

**Table 1:** Plant species potentially pollinated by *E*. *ensifera*. Data (mean corolla length, mean operative length, flowering period and distribution) collected from the literature, herbarium vouchers and personal field observations (source).

Plant species	Mean corolla length ± standard error [mm] (measured individuals)	Mean operative length^1^ ± standard error [mm](measured individuals)	Flowering period	Recorded countries	Source
Lamiaceae					
*Salvia dombeyi* Epling	90.42 ± 5.13 (*n* = 3)	117.65 ± 7.39 (*n* = 3)	From January to April	Bolivia, Peru	[39,40]; USM; field collections
Loranthaceae					
*Aetanthus dichotomus* (Ruiz & Pav.) Kuijt	84.54 ± 8.58 (n = 6)	110.32 ± 5.58 (n = 2)	July, Sept	Colombia, Ecuador, Peru	[41]; USM
*Aetanthus macranthus* (Hook.) Kuijt	140.96 ± 20.33 (n = 4)	173.46 ± 9.92 (n = 2)	-	Ecuador, Perú	MO
*Aetanthus mutisii* (Kunth) Engl.	102.50 ± 15.77 (n = 2)	135.67 ± 8.26 (n = 2)	-	Colombia	MO
*Tristerix grandiflorus* (Ruiz & Pav.) Barlow & Wiens	95.63 ± 7.60 (n = 3)	101.48 ± 4.16 (n = 3)	-	Ecuador, Peru	[30]; MO
Passifloraceae					
*Passiflora ampullacea* (Mast.) Harms	79.97 ± 12.24 (n = 11)	109.53 ± 14.9 (n = 11)	July to Sept	Ecuador	[35,36]; E; F; GH; K; NMNH
*Passiflora anastomosans* (Lamb. ex DC.) Killip	106.71 ± 3.93 (n = 3)	133.25 ± 3.66 (n = 3)	-	Peru	[36]; F
*Passiflora bracteosa Planch*. *& Liden ex Triana & Planch*.	78.64 ± 3.33 (n = 9)	97.51 ± 4.63 (n = 9)	-	Venezuela, Colombia	[37]; NMNH
*Passiflora coactilis* (Mast.) Killip	80.28 ± 4.56 (n = 7)	124.72 ± 4.18 (n = 7)	-	Colombia, Ecuador	[36]; E; F; NMNH
*Passiflora crispolanata L*. *Uribe*	62–84	-	-	Colombia	[37]
*Passiflora cumbalensis* (H. Karst.) Harms	90.05 ± 2.77 (n = 15)	118.66 ± 5.54 (n = 4)	All the year	Colombia, Ecuador, Peru	USM; [36]
*Passiflora lanata* (Juss.) Poir.	78.15 ± 3.19 (n = 8)	105.83 ± 2.92 (n = 8)	May to Nov	Colombia	[37]; NMNH
*Passiflora leptomischa* Harms	66.31 ± 10.45 (n = 4)	100.78 ± 10.53 (n = 4)	-	Colombia	[37]; K
*Passiflora loxensis* Killip & Cuatrec.	80.57 ± 3.79 (n = 3)	146 ± 2.70 (n = 3)	-	Ecuador	[35]; NMNH
*Passiflora mixta L*. *F*	82.76 ± 2.08 (n = 16)	110.39 ± 4.03 (n = 7)	June to Dec	Venezuela, Colombia, Ecuador, Peru, Bolivia	USM
*Passiflora parritae*(Mast.) L. H. Bailey	71.70 ± 5.56 (n = 3)	103.25 ± 5.97 (n = 3)	-	Colombia	[37]; F; K; NMNH
*Passiflora parvifolia* (DC.) Harms	72.98 ± 6.35 (n = 5)	95.59 ± 7.24 (n = 5)	-	Peru	[37]; E; F; NHMUK; NMNH
*Passiflora rugosa*(Mast.) Triana & Planch.	82.68 ± 3.68 (n = 10)	118.72 ± 7.09 (n = 10)	-	Colombia	[36]; F; K; NHMUK; NMNH
*Passsiflora sanctaebarbarae* Holm-Niels. & P. Jørg.	80–85	-	-	Ecuador	[35]
*Passiflora tenerifensis L*.*K*. *Escobar*	67–100	-	-	Colombia	[37]
*Passiflora trinervia* (Juss.) Poir.	109.04 ± 6.81 (n = 3)	116.55 ± 4.14 (n = 3)	-	Colombia	[37]; NMNH
*Passiflora tripartita* var. *mollisima* (Kunth) Holm-Niels. & P. Jørg.	86.12 ± 1.85 (n = 44)	112.94 ± 2.48 (n = 31)	All the year	Venezuela, Colombia, Ecuador, Peru, Bolivia	field collections; NY; USM
Solanaceae					
*Brugmansia sanguinea*(Ruiz & Pav.) D. Don	172.61 ± 3.7 (n = 15)	-	All the year	Venezuela, Colombia, Ecuador, Peru, Bolivia	USM
*Salpichroa didierana* Jaub.	111.99 ± 3.88 (n = 20)	143.14 ± 5.19 (n = 20)	Sept to Feb	Peru	field collections; USM; [38]

^1^Distance from anthers and stigma to nectary

### Flower and bill lengths

We measured corolla tube length and flower operative length (i.e. distance from anthers and stigma to nectary) of the 24 species. Measurements were taken from 2 to 44 individuals per plant species, either in the field or from voucher specimens deposited in different herbaria (Table 1). Bill length data of *E*. *ensifera* was obtained from 51 georeferenced museum records of male individuals published by Sánchez Osés [27]. Mean value of the corolla’ operative length of the guild of plants measured from herbarium vouchers (21 plant species, Table 1) was compared with bill length of 51 *E*. *ensifera* individuals by fitting a generalized linear model (GLM) with gamma error distribution and log link function in R [42]. The model consisted of length as the response variable and organism trait length (hummingbird bill and plant tube) as the fixed factor. In addition a null model with 5000 simulations was performed to calculate a pseudo-F which was compared with the observed F.

### Occurrence data

To reduce potential sampling bias, initial georeferenced points were spatially thinned; to this end, clusters were eliminated by keeping 4 km as the minimum distance between points [43]. Occurrence points for *E*. *ensifera*were obtained from the Global Biodiversity Information Facility (http://www.gbif.org/). If information source was ambiguous, occurrence points were discarded for greater reliability.

### Species distribution modeling and range polygons

Species distribution was modeled for *E*. *ensifera* using one topographic (altitude) and 19 bioclimatic variables obtained from WorldClim database (http://www.worldclim.org/bioclim) with a spatial resolution of 2.5 arc-min. SDM was performed from presence-only records via maximum entropy method using the program MAXENT v.3.3.3k [44]. Before deciding final MAXENT settings, we tested different preliminary models by changing default settings for each run [45]. Optimal prediction models were searched by modifying the extent of the prediction range [46]. Model performance was defined with the area under the curve (AUC) of the receiver operating characteristic (ROC) plot. The final resulting prediction area was located in the northeast region of South America (from Venezuela to Bolivia), from 12.347246 N to 23.014439 S and from 81.773802 W to 60.370377 E. Models were refined by alternatively changing the regularization multiplier to 0.5, 1, 1.5 or 2. Regularization penalizes each term included in the model, thus preventing overfitting [45]. In addition, autofeatures option was selected, which allows linear, quadratic, product, threshold, and hinge feature types to relate species records and environmental variables [47]. Background data was randomly selected by MAXENT from the prediction area using the default option which assumes that every pixel has the same probability of being selected as background [47]. The final settings used were: random test percentage = 25, regularization multiplier = 1, convergence threshold = 0.00001, and maximum iterations = 1,000. A total of 10 bootstrap replicates were generated for each model. A jackknife test was conducted to assess the relative importance of each one of the environmental variables in the MAXENT model. Only those variables that individually contributed more than 5% to the SDM were reported. Projections of the final SDM of MAXENT onto the geographical space were visualized and edited in QGIS v.2.14 [48]. The 10 percentile training presence threshold was used to visualize the predicted area onto the geographical space, thus excluding 10% of the presence points with the lowest predicted values.

Range polygons were obtained for 19 of the 24 plant species of the guild from the BIEN package of R [49]. Range polygons were not available for *S*. *dombeyi*, *A*. *mutisii*, *P*. *bracteosa*, *P*. *leptomischa*, and *P*. *tenerifensis*. Thus, these plant species were not included in the following analyses.

### Mosaic of overlapping ranges

The SDM of *E*. *ensifera* was converted into presence-absence maps considering the 10 percentile threshold. For the guild of plants, a map was generated by adding all the plant species range polygons (“richness map”). To quantify the areas of overlap between *E*. *ensifera* and its plant guild and reciprocally, i.e. between the plant guild and *E*. *ensifera*, the number of overlapped pixels on the resulting maps was counted and converted to km^2^. A visual representation of the number of plant species overlapping with *E*. *ensifera* along its range was generated by trimming the richness map to fit the SDM of *E*. *ensifera*.

To determine the relationship between bill length and plant richness we fitted a GLM with a gamma error distribution using the 51 bill length records of male hummingbird. We visualized the regressions using the ggplot2 package in R [50].

## Results

### Flower and bill lengths

**Fig 2:**
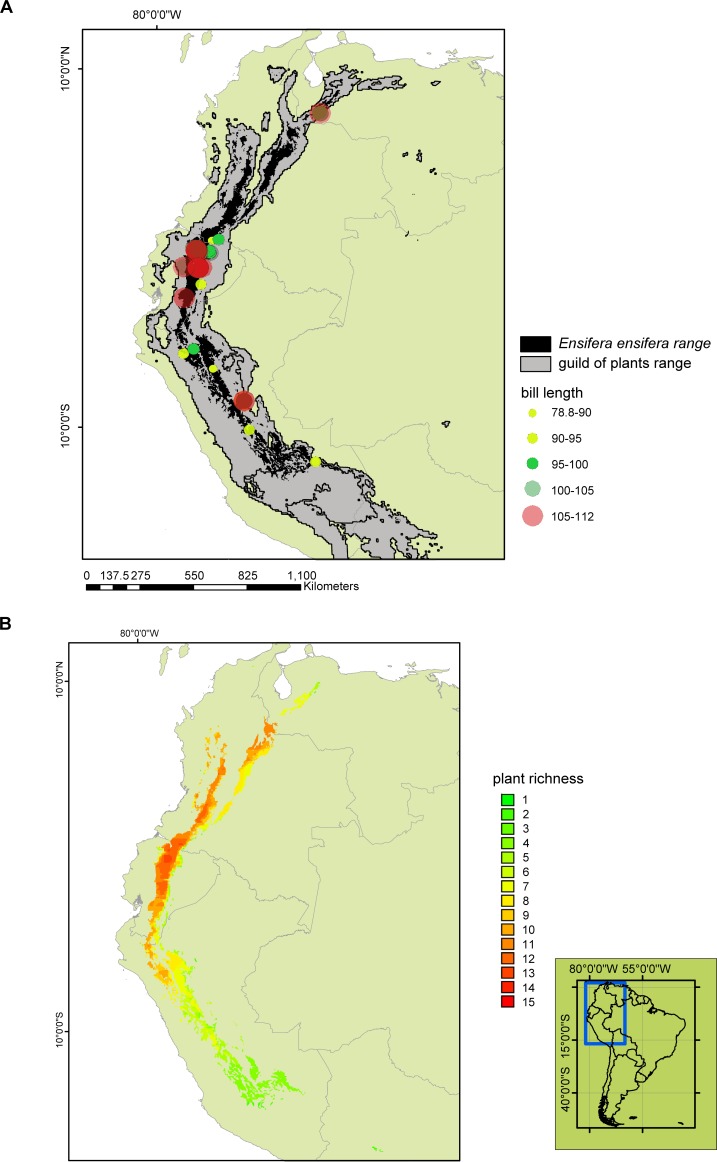
A, Predicted range overlap of *E*. *ensifera* and 19 long-flowered plant species. B, Richness of the long-flowered plant species within *E*. *ensifera* range. Background vector mapswere obtained from the public domain dataset Natural Earth @naturalearthdata.com.

**Fig 3:**
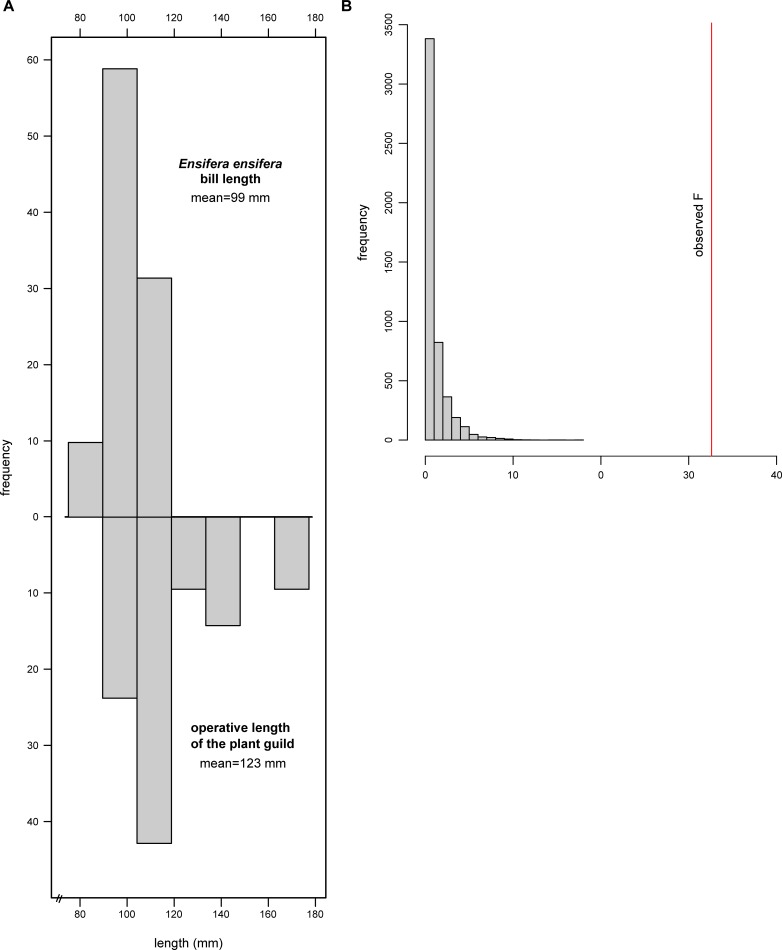
A, Histogram of the distribution of bill length (n = 51) and operative length of the long-flowered guild of plants (n = 21). B, Histogram of the distribution of pseudo-F values resulting from 5000 simulations of the linear model comparing mean of bill length and operative length of the long-flowered guild of plants; the solid line indicates the observed F value.

Mean operative length of the 24 species of the plant guild was between 95.59 ± 7.24 mm in *P*. *parvifolia* up to 146 ± 2.70 mm in *P*. *loxensis* (Table 1). Mean bill length of *E*. *ensifera* was 99.39 ± 13.92 mm and varied geographically, without showing a clear clinal pattern (Fig 2A). The longest bills were recorded at mid-latitudes of its distribution range, around the Ecuadorian Andes (Fig 2A). Mean operative length of the guild of plants was significantly longer than bill length (F = 32.61, P < 0.001; Fig 3). The observed F value was significantly different from the pseudo-F generated by the null model (P < 0.001), i.e. the observed pattern is not a product of chance (Fig 3B).

### Species distribution modeling

The SDM showed an AUC value of 0.97 ± 0.01. Altitude was the most important variable in contributing to the SDM, (Table 2). The boundary of the predicted distribution of *E*. *ensifera* was similar to that in other maps of the range of this species [27,51]. Predicted ranges of *P*. *tripartita*,*P*. *mixta*, and *B*. *sanguinea* were broader than *E*. *ensifera* range, extending considerably further to the south than the area predicted for the pollinator (Fig 2A and S2 Fig).

**Table 2:** Percentage contribution of the environmental variables to the species distribution model of *E*. *ensifera*.

Species and variables	Contribution (%)
*Ensifera ensifera*	
altitude	41.8
precipitation seasonality	11.6
mean temperature of the wettest quarter	10.4
mean temperature of the warmest quarter	7.6
min temperature of the coldest month	7.3
temperature seasonality	6.4

### Mosaic of overlapping range

The predicted range of *E*. *ensifera* covered an area of 256,547 km^2^, and was completely nested within that of the plant guild (1,729,836 km^2^; Fig 2A). In turn, the plant guild overlapped 21% of its distribution with that of *E*. *ensifera*. The sword-billed hummingbird overlapped with eight of the 19 plant species across most of its entire range, and with 12 at the center of its distribution (Fig 2A). Plant richness was highest at mid-latitudes and lowest in the south of *E*. *ensifera* range (Fig 2B).

**Fig 4:**
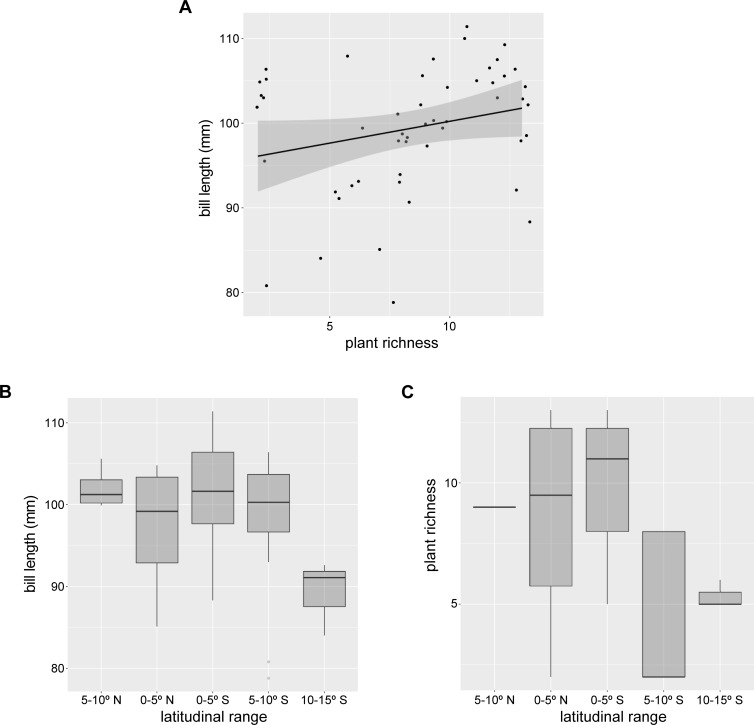
Relationship between 51 records of (A) bill length and (B) species richness of the long-flowered guild of plants with latitude. Shaded bands represent 95% confidence intervals around the solid line. Distribution of the values of (C) bill length and (D) richness across five latitudinal ranges.

The longest bills were detected near the Ecuadorian Andes, whereas the shortest bills were recorded in northern Peruvian Andes (Fig 2A). *Ensifera ensifera* bill length and guild of plant richness showed a positive trend (GLM: estimate = 0.01, t = 1.78, P = 0.08, Fig 4A). The longest bill lengths were detected in areas of high plant richness, i.e.: in the center of *E*. *ensifera* distribution, between 0 and 5° S (Fig 4B and 4C).

## Discussion

### Geographical ranges

The high geographical overlap between *E*. *ensifera* and its guild of long-flowered plants, together with the observed trait matching between hummingbird bill length and mean operative flower length, lends support to the existence of phenotypic specialization resulting from a coevolutionary process. This assumption is strengthened by the evidence of consistent phylogenetic divergence ages reported for *E*. *ensifera* (10.7 ma) and long-flowered species of *Passiflora* supersection Tacsonia (11.6 ma) [23,52]. In addition, as a first geographical insight into this interaction system, our results reinforce the developing concept of interactions occurring in a geographically structured mosaic across the landscape. The geographical structuring of the interaction shows potential variation in the size and degree of asymmetry of the interaction networks, since the ranges of plant species did not completely overlap with each other and with the range of *E*. *ensifera*. A similar pattern was also evidenced in the pollination mutualism between the extremely curved-billed *Eutoxeres* hummingbirds and their matching plant species [53].

### Trait matching

Previous studies suggest that reciprocal selection drives escalated lengthening of interacting traits of plant and insects [2]. Although this assumption needs to be confirmed for flowers and bills in hummingbird-plant interactions, there is evidence suggesting that this may indeed be the case. On the one hand, selection driven co-adaptation has been demonstrated for flower and bill morphology [53]. On the other hand, studies in plant-hummingbird networks have shown that long matching traits benefit both sides of the relationship [19,20]. Since hummingbirds with the longest bills will sip the most nectar from long flowers, and deepest flowers will be more often pollinated, then conditions are met for reciprocally driven positive directional selection and hence for an escalated lengthening of both bills and flowers [3,54,55]. For the *E*. *ensifera* guild, mean operative flower length was greater than bill length, as expected for morphological complementarity in coadapted plant-pollinator relationships (see [56]).

### Ecological specialization

Extreme one-to-one specialization is rare in plant-pollinator systems, since the flower resources exploited by the nectar-feeding visitors are ephemeral, rendering the dependence on a single plant species unreliable. Even in the highly specialized interaction between the long-tongued hawkmoth *Xantopham morganii praedicta* and the long-spurred orchid *A*. *sesquipedale*, other long-spurred species have been proposed as possible food plants [3]. Plant-pollinator interactions most likely occur as a broad network of interactions [14], with long-billed hummingbirds acquiring specialization by more frequently interacting with flowers that match in length than with shorter ones [19]. Our results suggest that the sword-billed hummingbird locally relies on several long-flowered nectar sources, since at least three plant species simultaneously overlapped with the hummingbird distribution range. *Ensifera ensifera* bill length varied across the geographical range, possibly creating differences in the degree of asymmetry of interactions across the landscape, as expected under a geographical mosaic of coevolution [28].

### Hotspots and coevolutionary vortices

We detected an area where species are predicted to participate in a “coevolutionary vortex” at mid-latitudes of *E*. *ensifera* distribution. There, *E*. *ensifera* exhibited the longest bills, probably acquired by escalated reciprocal selection, which presumably drew several species to converge matching traits. We cannot ascertain whether escalation, if present, was driven by pairwise interaction and later increased in the number of interacting species, or if the joint action of the plant guild has driven coevolution in a diffuse manner. Both possibilities are suggested by the high richness of the plant community in these areas. Despite a non-significant relationship, probably due to the small sample size, we observed a positive trend between bill length and species plant richness. Accordingly, a study on the relationship between proboscides length of a long-tongued fly and the long-flowered guild it pollinates showed that morphological traits of pollinators were more exaggerated at sites where the local plant community consisted of several species with even longer tubes than the proboscides [56]. However, information about the degree of asymmetry of the interaction networks of these communities is lacking.

### Coldspots

In a geographical context, the coevolutionary scenario in some communities may be intermingled with other communities in which selection is one-sided or absent [2]. For instance, at sites where the bills of *E*. *ensifera* are much shorter than floral tubes, unilateral selection of bills is the most likely scenario, since the hummingbirds with the longest bills will be favored. We expect this to happen in the south of the *E*. *ensifera* range, where bills are short (mean = 96.91 mm) and plant richness is low. In these areas, *P*. *tripartita*, *P*. *mixta* and *B*. *sanguinea* overlapped with *E*. *ensifera*, and their flower tubes are much longer (mean = 113, 110, and 173 mm, respectively) than the bill length. Based on these facts, we postulate that these areas represent coevolutionary coldspots. When both hummingbird and flower match at low trait magnitudes, we expect an incipient hotspot since we assume that escalated reciprocal selection will likely shape long complementary traits over time. Other coldspots are represented by areas where *E*. *ensifera* was absent within the range of the plant guild. Plants might colonize other areas outside the range of this hummingbird, where abiotic conditions are favorable, via alternative modes of reproduction (e.g. cultivation for ornamental purposes).

## Conclusions

Our results using SDMs suggest a geographical structuring of the relationship between the sword-billed hummingbird and its guild of long-flowered plants. We observed a pattern expected under a geographical mosaic scenario where the overlapping ranges of interacting species varied across the landscape. On the one hand, we observed a possible coevolutionary vortex area at mid-latitudes within *E*. *ensifera* range, where the whole plant guild overlapped with the hummingbird and where its bill was longest. On the other hand, we detected presumptive coevolutionary coldspots where the hummingbird either has short bill or is absent. Our study provides an approach to the geographical dimension of coevolution in this phenotypically specialized plant-pollinator system. It is important to note that other selective forces than those exerted by flower-operative length could be determining the longest bills attained in the mid-latitudes of hummingbird distribution. For instance, long bills could reflect a selection to reduce interspecific competition for food resource [57]. Meanwhile, in related to the interacting counterpart, high plant richness variation may not be necessary forced by longest bills selection pressure but instead being a latitudinal pattern [58]. Therefore, further experimental studies focused on confirming the actual composition and topology of the interaction networks and the reciprocal dependence and selection of the interacting partners should be carried out to thoroughly test the coevolutionary hypothesis.

## Supporting information

S1 Fig **Representative plant species pollinated by the sword-billed hummingbird *Ensifera ensifera*: (a) *Aetanthus dichotomus* (Lorantaceae), (b) *Brugmansia sanguinea* (Solanaceae), (c) *Salvia dombeyi* (Lamiaceae), (d) *Passiflora mixta* (Passifloraceae), (e) *P*. *tripartita* var. *mollisima* (Passifloraceae), (f) *P*. *cumbalensis (*Passifloraceae), and (g) *Salpichroa didierana* (Solanaceae).**Photo credits: (a) A. Kay*, (b) and (e) S. Leiva González, (c) and (g) A.A. Cocucci, d) L. Agudelo*, and (f) R. Culbert*. Scale bars equal to 1 cm. * Photo shared by Flickr.com.
(EPS)

S2 Fig Potential current ranges of the sword-billed hummingbird *E*. *ensifera*. Probability of occurrence averaged after 10 cross-validation runs at the potential suitable habitat based on the SDMs is indicated with yellow to red colors. Background vector maps of was obtained from the public domain dataset Natural Earth @naturalearthdata.com.
(EPS)

S1 Table Occurrence data collected for the sword-billed hummingbird *E*. *ensifera*. (DOCX)
