# Correction: Range overlap between the sword-billed hummingbird and its guild of long-flowered species: An approach to the study of a coevolutionary mosaic

**DOI:** 10.1371/journal.pone.0213036

**Published:** 2019-02-22

**Authors:** Florencia Soteras, Marcela Moré, Ana C. Ibañez, María del Rosario Iglesias, Andrea A. Cocucci

A column containing the GBIF occurrence URLs is missing from S1 Table. Please view the correct S1 Table below.

## Supporting information

S1 Table Occurrence data collected for the sword-billed hummingbird *E*. *ensifera*. (DOCX)
