# Supplementary material for: Range overlap between the sword-billed hummingbird and its guild of long-flowered species: An approach to the study of a coevolutionary mosaic
Source: PLoS One. 2018 Dec 26;13(12):e0209742. doi: 10.1371/journal.pone.0209742 (PMC6306261; doi:10.1371/journal.pone.0209742)
Supplement: S1 Table — (DOCX) [file pone.0209742.s003.docx]

**Table S1.** Occurrence data collected for the sword-billed hummingbird *E. ensifera* and the 11 species of the plant guild modelled in this study.

| species | latitude | longitude |
| --- | --- | --- |
| **Sword-billed hummingbird** |  |  |
| *Ensifera ensifera* | -15.152 | -68.972 |
| *Ensifera ensifera* | -13.595833 | -72.879166 |
| *Ensifera ensifera* | -13.57825 | -72.88536 |
| *Ensifera ensifera* | -13.571195 | -72.89136 |
| *Ensifera ensifera* | -13.320807 | -71.59653 |
| *Ensifera ensifera* | -13.309302 | -72.045166 |
| *Ensifera ensifera* | -13.251652 | -72.43179 |
| *Ensifera ensifera* | -13.221036 | -72.38796 |
| *Ensifera ensifera* | -13.175019 | -71.58786 |
| *Ensifera ensifera* | -13.158254 | -71.631546 |
| *Ensifera ensifera* | -13.133333 | -71.416664 |
| *Ensifera ensifera* | -13.068846 | -72.39859 |
| *Ensifera ensifera* | -13.0643835 | -71.53962 |
| *Ensifera ensifera* | -12.9626 | -73.64027 |
| *Ensifera ensifera* | -12.784994 | -73.9952 |
| *Ensifera ensifera* | -11.767342 | -75.166306 |
| *Ensifera ensifera* | -11.717231 | -75.074814 |
| *Ensifera ensifera* | -11.566277 | -74.94246 |
| *Ensifera ensifera* | -11.499538 | -74.86496 |
| *Ensifera ensifera* | -11.4693 | -74.7956 |
| *Ensifera ensifera* | -11.405322 | -74.65759 |
| *Ensifera ensifera* | -9.87417 | -76.42155 |
| *Ensifera ensifera* | -9.779438 | -76.089935 |
| *Ensifera ensifera* | -9.737314 | -76.16984 |
| *Ensifera ensifera* | -9.695173 | -76.08676 |
| *Ensifera ensifera* | -7.003278 | -78.19889 |
| *Ensifera ensifera* | -6.909503 | -77.88487 |
| *Ensifera ensifera* | -6.860133 | -77.70244 |
| *Ensifera ensifera* | -6.8398676 | -77.785866 |
| *Ensifera ensifera* | -6.8168516 | -77.94679 |
| *Ensifera ensifera* | -6.7823668 | -77.890144 |
| *Ensifera ensifera* | -6.7348576 | -77.82535 |
| *Ensifera ensifera* | -6.694833 | -77.69067 |
| *Ensifera ensifera* | -6.4197073 | -77.92362 |
| *Ensifera ensifera* | -6.3259063 | -78.20128 |
| *Ensifera ensifera* | -6.3016486 | -78.00636 |
| *Ensifera ensifera* | -6.2661576 | -77.91229 |
| *Ensifera ensifera* | -5.906336 | -78.42762 |
| *Ensifera ensifera* | -5.820069 | -78.01349 |
| *Ensifera ensifera* | -5.8101506 | -78.02101 |
| *Ensifera ensifera* | -5.6958833 | -77.807465 |
| *Ensifera ensifera* | -4.1094785 | -78.96595 |
| *Ensifera ensifera* | -4.0422077 | -79.14879 |
| *Ensifera ensifera* | -3.6682982 | -79.24816 |
| *Ensifera ensifera* | -2.844617 | -79.14274 |
| *Ensifera ensifera* | -2.7935398 | -79.209366 |
| *Ensifera ensifera* | -2.5439522 | -78.8914 |
| *Ensifera ensifera* | -1.9002863 | -78.17871 |
| *Ensifera ensifera* | -1.6051672 | -78.63979 |
| *Ensifera ensifera* | -1.3977 | -78.42796 |
| *Ensifera ensifera* | -0.6769595 | -77.59884 |
| *Ensifera ensifera* | -0.658633 | -78.374626 |
| *Ensifera ensifera* | -0.6229469 | -77.838135 |
| *Ensifera ensifera* | -0.5710266 | -77.7612 |
| *Ensifera ensifera* | -0.5409831 | -77.88182 |
| *Ensifera ensifera* | -0.4987787 | -78.24657 |
| *Ensifera ensifera* | -0.4617624 | -77.899025 |
| *Ensifera ensifera* | -0.4558442 | -78.317795 |
| *Ensifera ensifera* | -0.4397921 | -78.49903 |
| *Ensifera ensifera* | -0.4094089 | -78.365906 |
| *Ensifera ensifera* | -0.3776523 | -78.16137 |
| *Ensifera ensifera* | -0.3579116 | -78.15159 |
| *Ensifera ensifera* | -0.3291159 | -78.209526 |
| *Ensifera ensifera* | -0.2789057 | -78.25029 |
| *Ensifera ensifera* | -0.2059932 | -78.52409 |
| *Ensifera ensifera* | -0.1778409 | -78.599106 |
| *Ensifera ensifera* | -0.165653 | -78.58246 |
| *Ensifera ensifera* | -0.1343446 | -78.53408 |
| *Ensifera ensifera* | -0.1253127 | -78.57044 |
| *Ensifera ensifera* | -0.1117873 | -78.584885 |
| *Ensifera ensifera* | -0.0681496 | -78.60744 |
| *Ensifera ensifera* | -0.0477219 | -78.63327 |
| *Ensifera ensifera* | -0.0057507 | -78.682106 |
| *Ensifera ensifera* | 0.008248 | -78.546326 |
| *Ensifera ensifera* | 0.02661 | -78.55612 |
| *Ensifera ensifera* | 0.0338137 | -78.48593 |
| *Ensifera ensifera* | 0.0370789 | -78.480835 |
| *Ensifera ensifera* | 0.0782042 | -78.531265 |
| *Ensifera ensifera* | 0.1035 | -78.5306 |
| *Ensifera ensifera* | 0.1148667 | -78.58362 |
| *Ensifera ensifera* | 0.1167 | -78.7667 |
| *Ensifera ensifera* | 0.2338254 | -78.26281 |
| *Ensifera ensifera* | 0.6050736 | -77.701035 |
| *Ensifera ensifera* | 0.8274867 | -77.59689 |
| *Ensifera ensifera* | 1.2116673 | -77.279205 |
| *Ensifera ensifera* | 1.9166666 | -76.833336 |
| *Ensifera ensifera* | 2.0594928 | -76.343994 |
| *Ensifera ensifera* | 2.125 | -76.43633 |
| *Ensifera ensifera* | 2.3016667 | -76.240005 |
| *Ensifera ensifera* | 2.5333333 | -76.95 |
| *Ensifera ensifera* | 3.5666666 | -76.583336 |
| *Ensifera ensifera* | 3.5798306 | -76.5721 |
| *Ensifera ensifera* | 4.383333 | -74.316666 |
| *Ensifera ensifera* | 4.468917 | -75.54182 |
| *Ensifera ensifera* | 4.4774504 | -75.28376 |
| *Ensifera ensifera* | 4.485344 | -73.89421 |
| *Ensifera ensifera* | 4.5333333 | -73.933334 |
| *Ensifera ensifera* | 4.599824 | -74.06037 |
| *Ensifera ensifera* | 4.630313 | -74.00919 |
| *Ensifera ensifera* | 4.6455946 | -74.04339 |
| *Ensifera ensifera* | 4.7357144 | -73.92623 |
| *Ensifera ensifera* | 4.759536 | -74.017426 |
| *Ensifera ensifera* | 5.083 | -75.41783 |
| *Ensifera ensifera* | 5.5416064 | -75.80336 |
| *Ensifera ensifera* | 6.348846 | -76.174164 |
| *Ensifera ensifera* | 6.415954 | -76.08135 |
| *Ensifera ensifera* | 6.433695 | -76.07535 |
| *Ensifera ensifera* | 8.85818 | -70.70131 |
