# Supplementary material for: Correction: Range overlap between the sword-billed hummingbird and its guild of long-flowered species: An approach to the study of a coevolutionary mosaic
Source: PLoS One. 2019 Feb 22;14(2):e0213036. doi: 10.1371/journal.pone.0213036 (PMC6386474; doi:10.1371/journal.pone.0213036)
Supplement: S1 Table — (DOCX) [file pone.0213036.s001.docx]

**Table S1.** Occurrence data collected for the sword-billed hummingbird *E. ensifera* and the 11 species of the plant guild modelled in this study.

| species | latitude | longitude | GBIF Occurrence URL |
| --- | --- | --- | --- |
| **Sword-billed hummingbird** |  |  |  |
| *Ensifera ensifera* | -15.152 | -68.972 | https://www.gbif.org/occurrence/587524722 |
| *Ensifera ensifera* | -13.595833 | -72.879166 | https://www.gbif.org/occurrence/262340528 |
| *Ensifera ensifera* | -13.57825 | -72.88536 | https://www.gbif.org/occurrence/588979220 |
| *Ensifera ensifera* | -13.571195 | -72.89136 | https://www.gbif.org/occurrence/148865950 |
| *Ensifera ensifera* | -13.320807 | -71.59653 | https://www.gbif.org/occurrence/588322968 |
| *Ensifera ensifera* | -13.309302 | -72.045166 | https://www.gbif.org/occurrence/261919903 |
| *Ensifera ensifera* | -13.251652 | -72.43179 | https://www.gbif.org/occurrence/829195361 |
| *Ensifera ensifera* | -13.221036 | -72.38796 | https://www.gbif.org/occurrence/257072319 |
| *Ensifera ensifera* | -13.175019 | -71.58786 | https://www.gbif.org/occurrence/588340965 |
| *Ensifera ensifera* | -13.158254 | -71.631546 | https://www.gbif.org/occurrence/262958609 |
| *Ensifera ensifera* | -13.133333 | -71.416664 | https://www.gbif.org/occurrence/588002039 |
| *Ensifera ensifera* | -13.068846 | -72.39859 | https://www.gbif.org/occurrence/830302083 |
| *Ensifera ensifera* | -13.0643835 | -71.53962 | https://www.gbif.org/occurrence/829668266 |
| *Ensifera ensifera* | -12.9626 | -73.64027 | https://www.gbif.org/occurrence/260538313 |
| *Ensifera ensifera* | -12.784994 | -73.9952 | https://www.gbif.org/occurrence/180012580 |
| *Ensifera ensifera* | -11.767342 | -75.166306 | https://www.gbif.org/occurrence/587883927 |
| *Ensifera ensifera* | -11.717231 | -75.074814 | https://www.gbif.org/occurrence/589186974 |
| *Ensifera ensifera* | -11.566277 | -74.94246 | https://www.gbif.org/occurrence/587883754 |
| *Ensifera ensifera* | -11.499538 | -74.86496 | https://www.gbif.org/occurrence/588318376 |
| *Ensifera ensifera* | -11.4693 | -74.7956 | https://www.gbif.org/occurrence/148865900 |
| *Ensifera ensifera* | -11.405322 | -74.65759 | https://www.gbif.org/occurrence/588181249 |
| *Ensifera ensifera* | -9.87417 | -76.42155 | https://www.gbif.org/occurrence/829565858 |
| *Ensifera ensifera* | -9.779438 | -76.089935 | https://www.gbif.org/occurrence/588340311 |
| *Ensifera ensifera* | -9.737314 | -76.16984 | https://www.gbif.org/occurrence/588334076 |
| *Ensifera ensifera* | -9.695173 | -76.08676 | https://www.gbif.org/occurrence/180012789 |
| *Ensifera ensifera* | -7.003278 | -78.19889 | https://www.gbif.org/occurrence/588346309 |
| *Ensifera ensifera* | -6.909503 | -77.88487 | https://www.gbif.org/occurrence/830669782 |
| *Ensifera ensifera* | -6.860133 | -77.70244 | https://www.gbif.org/occurrence/262941630 |
| *Ensifera ensifera* | -6.8398676 | -77.785866 | https://www.gbif.org/occurrence/830373984 |
| *Ensifera ensifera* | -6.8168516 | -77.94679 | https://www.gbif.org/occurrence/829668549 |
| *Ensifera ensifera* | -6.7823668 | -77.890144 | https://www.gbif.org/occurrence/829656781 |
| *Ensifera ensifera* | -6.7348576 | -77.82535 | https://www.gbif.org/occurrence/829668152 |
| *Ensifera ensifera* | -6.694833 | -77.69067 | https://www.gbif.org/occurrence/829662890 |
| *Ensifera ensifera* | -6.4197073 | -77.92362 | https://www.gbif.org/occurrence/587948246 |
| *Ensifera ensifera* | -6.3259063 | -78.20128 | https://www.gbif.org/occurrence/830534069 |
| *Ensifera ensifera* | -6.3016486 | -78.00636 | https://www.gbif.org/occurrence/589196663 |
| *Ensifera ensifera* | -6.2661576 | -77.91229 | https://www.gbif.org/occurrence/589186803 |
| *Ensifera ensifera* | -5.906336 | -78.42762 | https://www.gbif.org/occurrence/261925941 |
| *Ensifera ensifera* | -5.820069 | -78.01349 | https://www.gbif.org/occurrence/589197760 |
| *Ensifera ensifera* | -5.8101506 | -78.02101 | https://www.gbif.org/occurrence/830368063 |
| *Ensifera ensifera* | -5.6958833 | -77.807465 | https://www.gbif.org/occurrence/588341593 |
| *Ensifera ensifera* | -4.1094785 | -78.96595 | https://www.gbif.org/occurrence/260473721 |
| *Ensifera ensifera* | -4.0422077 | -79.14879 | https://www.gbif.org/occurrence/829029496 |
| *Ensifera ensifera* | -3.6682982 | -79.24816 | https://www.gbif.org/occurrence/588309521 |
| *Ensifera ensifera* | -2.844617 | -79.14274 | https://www.gbif.org/occurrence/830351148 |
| *Ensifera ensifera* | -2.7935398 | -79.209366 | https://www.gbif.org/occurrence/262929891 |
| *Ensifera ensifera* | -2.5439522 | -78.8914 | https://www.gbif.org/occurrence/830367891 |
| *Ensifera ensifera* | -1.9002863 | -78.17871 | https://www.gbif.org/occurrence/780077024 |
| *Ensifera ensifera* | -1.6051672 | -78.63979 | https://www.gbif.org/occurrence/588323468 |
| *Ensifera ensifera* | -1.3977 | -78.42796 | https://www.gbif.org/occurrence/589086419 |
| *Ensifera ensifera* | -0.6769595 | -77.59884 | https://www.gbif.org/occurrence/830107282 |
| *Ensifera ensifera* | -0.658633 | -78.374626 | https://www.gbif.org/occurrence/587339066 |
| *Ensifera ensifera* | -0.6229469 | -77.838135 | https://www.gbif.org/occurrence/829130352 |
| *Ensifera ensifera* | -0.5710266 | -77.7612 | https://www.gbif.org/occurrence/830586479 |
| *Ensifera ensifera* | -0.5409831 | -77.88182 | https://www.gbif.org/occurrence/829582803 |
| *Ensifera ensifera* | -0.4987787 | -78.24657 | https://www.gbif.org/occurrence/589210802 |
| *Ensifera ensifera* | -0.4617624 | -77.899025 | https://www.gbif.org/occurrence/587881640 |
| *Ensifera ensifera* | -0.4558442 | -78.317795 | https://www.gbif.org/occurrence/830505384 |
| *Ensifera ensifera* | -0.4397921 | -78.49903 | https://www.gbif.org/occurrence/829321182 |
| *Ensifera ensifera* | -0.4094089 | -78.365906 | https://www.gbif.org/occurrence/780075942 |
| *Ensifera ensifera* | -0.3776523 | -78.16137 | https://www.gbif.org/occurrence/588015814 |
| *Ensifera ensifera* | -0.3579116 | -78.15159 | https://www.gbif.org/occurrence/829413466 |
| *Ensifera ensifera* | -0.3291159 | -78.209526 | https://www.gbif.org/occurrence/829519355 |
| *Ensifera ensifera* | -0.2789057 | -78.25029 | https://www.gbif.org/occurrence/830249080 |
| *Ensifera ensifera* | -0.2059932 | -78.52409 | https://www.gbif.org/occurrence/260567235 |
| *Ensifera ensifera* | -0.1778409 | -78.599106 | https://www.gbif.org/occurrence/780075920 |
| *Ensifera ensifera* | -0.165653 | -78.58246 | https://www.gbif.org/occurrence/589235894 |
| *Ensifera ensifera* | -0.1343446 | -78.53408 | https://www.gbif.org/occurrence/587882527 |
| *Ensifera ensifera* | -0.1253127 | -78.57044 | https://www.gbif.org/occurrence/148865736 |
| *Ensifera ensifera* | -0.1117873 | -78.584885 | https://www.gbif.org/occurrence/589203736 |
| *Ensifera ensifera* | -0.0681496 | -78.60744 | https://www.gbif.org/occurrence/830393561 |
| *Ensifera ensifera* | -0.0477219 | -78.63327 | https://www.gbif.org/occurrence/588322508 |
| *Ensifera ensifera* | -0.0057507 | -78.682106 | https://www.gbif.org/occurrence/257405774 |
| *Ensifera ensifera* | 0.008248 | -78.546326 | https://www.gbif.org/occurrence/828991019 |
| *Ensifera ensifera* | 0.02661 | -78.55612 | https://www.gbif.org/occurrence/830608338 |
| *Ensifera ensifera* | 0.0338137 | -78.48593 | https://www.gbif.org/occurrence/589222141 |
| *Ensifera ensifera* | 0.0370789 | -78.480835 | https://www.gbif.org/occurrence/589195423 |
| *Ensifera ensifera* | 0.0782042 | -78.531265 | https://www.gbif.org/occurrence/589232108 |
| *Ensifera ensifera* | 0.1035 | -78.5306 | https://www.gbif.org/occurrence/829416590 |
| *Ensifera ensifera* | 0.1148667 | -78.58362 | https://www.gbif.org/occurrence/829328798 |
| *Ensifera ensifera* | 0.1167 | -78.7667 | http://data.gbif.org/occurrences/352388914/ |
| *Ensifera ensifera* | 0.2338254 | -78.26281 | https://www.gbif.org/occurrence/589241839 |
| *Ensifera ensifera* | 0.6050736 | -77.701035 | https://www.gbif.org/occurrence/588019321 |
| *Ensifera ensifera* | 0.8274867 | -77.59689 | https://www.gbif.org/occurrence/828921818 |
| *Ensifera ensifera* | 1.2116673 | -77.279205 | https://www.gbif.org/occurrence/780077858 |
| *Ensifera ensifera* | 1.9166666 | -76.833336 | http://data.gbif.org/occurrences/453106524 |
| *Ensifera ensifera* | 2.0594928 | -76.343994 | https://www.gbif.org/occurrence/828824416 |
| *Ensifera ensifera* | 2.125 | -76.43633 | https://www.gbif.org/occurrence/828829405 |
| *Ensifera ensifera* | 2.3016667 | -76.240005 | https://www.gbif.org/occurrence/780075937 |
| *Ensifera ensifera* | 2.5333333 | -76.95 | http://data.gbif.org/occurrences/453106500 |
| *Ensifera ensifera* | 3.5666666 | -76.583336 | https://www.gbif.org/occurrence/589193466 |
| *Ensifera ensifera* | 3.5798306 | -76.5721 | https://www.gbif.org/occurrence/591079134 |
| *Ensifera ensifera* | 4.383333 | -74.316666 | http://data.gbif.org/occurrences/243830469 |
| *Ensifera ensifera* | 4.468917 | -75.54182 | https://www.gbif.org/occurrence/589237950 |
| *Ensifera ensifera* | 4.4774504 | -75.28376 | https://www.gbif.org/occurrence/828917360 |
| *Ensifera ensifera* | 4.485344 | -73.89421 | https://www.gbif.org/occurrence/780075929 |
| *Ensifera ensifera* | 4.5333333 | -73.933334 | http://data.gbif.org/occurrences/453106499 |
| *Ensifera ensifera* | 4.599824 | -74.06037 | https://www.gbif.org/occurrence/830677947 |
| *Ensifera ensifera* | 4.630313 | -74.00919 | https://www.gbif.org/occurrence/589243042 |
| *Ensifera ensifera* | 4.6455946 | -74.04339 | https://www.gbif.org/occurrence/259039731 |
| *Ensifera ensifera* | 4.7357144 | -73.92623 | https://www.gbif.org/occurrence/589202787 |
| *Ensifera ensifera* | 4.759536 | -74.017426 | https://www.gbif.org/occurrence/180012736 |
| *Ensifera ensifera* | 5.083 | -75.41783 | https://www.gbif.org/occurrence/828914232 |
| *Ensifera ensifera* | 5.5416064 | -75.80336 | https://www.gbif.org/occurrence/830285007 |
| *Ensifera ensifera* | 6.348846 | -76.174164 | https://www.gbif.org/occurrence/829654673 |
| *Ensifera ensifera* | 6.415954 | -76.08135 | https://www.gbif.org/occurrence/589243817 |
| *Ensifera ensifera* | 6.433695 | -76.07535 | https://www.gbif.org/occurrence/830300911 |
| *Ensifera ensifera* | 8.85818 | -70.70131 | https://www.gbif.org/occurrence/262138822 |
